# Investigations on Grating-Enhanced Waveguides for Wide-Angle Light Couplings

**DOI:** 10.3390/nano12223991

**Published:** 2022-11-12

**Authors:** Yitong Gu, Ning Wang, Haorui Shang, Fei Yu, Lili Hu

**Affiliations:** 1 Hangzhou Institute for Advanced Study, University of Chinese Academy of Sciences, No.1, Sub-Lane Xiangshan, Xihu District, Hangzhou 310024, China; 2 Shanghai Institute of Optics and Fine Mechanics, Chinese Academy of Sciences, Shanghai 201800, China; 3 Laboratory of Gravitational Wave Precision Measurement of Zhejiang Province, No.1, Sub-Lane Xiangshan, Xihu District, Hangzhou 310024, China; 4 Taiji Laboratory for Gravitational Wave Universe, No.1, Sub-Lane Xiangshan, Xihu District, Hangzhou 310024, China

**Keywords:** light coupling, waveguides, gratings, energy collection

## Abstract

As a universal physical scheme, effective light couplings to waveguides favor numerous applications. However, the low coupling efficiency at wide angles prohibits this fundamental functionality and thus lowers the performance levels of photonic systems. As previously found, the transmission gratings patterned on waveguide facets could significantly improve the large-angle-inputted efficiency to the order of 10${}^{-1}$. Here, we continue this study with a focus on a common scenario, i.e., a grating-modified waveguide excited by the Gaussian beam. A simplified 2D theoretical model is firstly introduced, proving that the efficiency lineshape could be well flattened by elaborately arranged diffractive gratings. For demonstration, subsequent explorations for proper grating geometries were conducted, and four structural configurations were selected for later full-wave numerical simulations. The last comparison studies showcase that the analytical method approximates the finite element method-based modelings. Both methods highlight grating-empowered coupling efficiencies, being 2.5 bigger than the counterparts of the previously reported seven-ring structure. All in all, our research provides instructions to simulate grating effects on the waveguide’s light-gathering abilities. Together with algorithm-designed coupling structures, it would be of great interest to further benefit real applications, such as bioanalytical instrumentation and quantum photon probes.

## 1. Introduction

The rapid developments in waveguide optics are of tremendous profit to scientific and industrial fields [1,2,3,4]. Especially in recent days, novel concepts such as hybrid waveguides [5], plasmonic waveguides [6,7], and meta-waveguides [8] have received growing interest. Among all, a common and vital optical scenario is to couple incoming light to the waveguide facet [9,10,11]. Highly efficient couplings would be particularly beneficial to emerging applications, such as bioanalytical instrumentation [12], medical diagnosis [13,14], and quantum photon-counting probes [15]. However, owing to the limited numerical aperture (NA), the low coupling coefficient of the standard waveguide (e.g., single mode fiber 28, SMF-28) fails to satisfy the above usage requirements. Particularly for the angle of θ > 20${}^{\circ }$, the efficiency of an SMF-28 (NA ≈ 0.14 at 1550 nm) is commonly below the order of 10${}^{-6}$ [16]. Thereby, upgrading waveguides’ broad-angle light collections has become increasingly crucial.

To address this difficulty, a series of concepts, including microlens [17], GRIN components [18], and metastructures [19], have been developed so as to promote waveguide endfaces’ couplings. Concerning wide-angle light collections, we have demonstrated that plasmonic nanoarray-enhanced fibers exhibit orders of efficiency enhancements [16]. The operating angles are extended up to 85° within a broadband spectral range (i.e., from 550 nm to 1650 nm) [20]. By integrating a set of well-planned dielectric rings, the experimental efficiency at 1550 nm reaches a maximum of 14.2% at an angle of 73${}^{\circ }$ [19].

In this report, we continue studying grating-modified waveguides to inspect their structure-dependent coupling efficiencies. Here we concentrate on transmission gratings consisting of dielectric (i.e., Si${}_{3}$N${}_{4}$) materials. Thanks to the high refractive index (RI) and low Ohmic loss, dielectric microstructures enable efficient power distribution at wanted diffraction orders [19,21]. More general discussions on grating performance can be found in some related literature [22,23,24]. At first, a simplified 2D model describing a Gaussian beam coupled to the waveguide is introduced, where the grating modifications could smooth the efficiency curves. After that, the binary-shaped grating, by altering its pitch, filling factor (FF), and height, is investigated in order to create high diffraction efficiencies. Last, the complete numerical simulations on grating-empowered waveguides are displayed via finite-element methods (FEM). In summary, the theoretical model alongside grating designs shows a practical path to boosting coupling efficiencies, thereby easing waveguide-coupling usage at large angle inputs.

## 2. Concept of Grating-Assisted Waveguide Couplings

**Figure 1:**
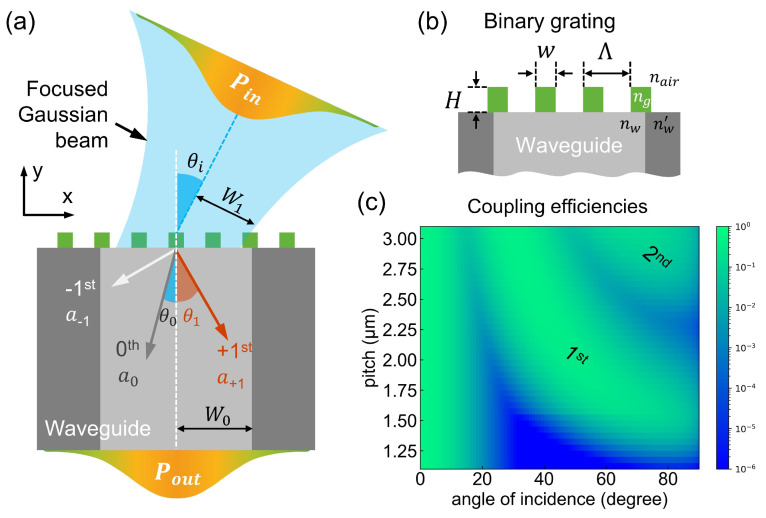
Grating-enhanced waveguides for wide-angle light collections. (**a**) Schematic showing a microstructure-modified waveguide excited by a focused Gaussian beam. (**b**) A close look at a binary grating. (**c**) The calculated coupling efficiencies based on a 2D analytical model.

The concept of grating-enabled waveguides is outlined in Figure 1. In (a), a dielectric grating (Si${}_{3}$N${}_{4}$, RI: 1.94, the pitch: Λ) is placed on the endface of a core-cladding waveguide (SiO${}_{2}$, RI: 1.466, the inner core width: $W_{0}$); a focused Gaussian beam (wavelength: λ = 1.55 μm, wavenumber: $k_{0}$ = 2π/λ, radius: $W_{1}$) impacts this surface at an angle of $\theta_{i}$. For simplicity, an ideal coupling scene is considered: the beam waist is located just at the grating, and there are no x- and y-axis misalignments between the beam, the grating, and the waveguide. Consequently, the coupling efficiency η($\theta_{i}$) is defined as η($\theta_{i}$) = $P_{out}/P_{in}$, where $P_{out}$ and $P_{in}$ represent the waveguide output and input powers, respectively.

As shown in Figure 1a, the transmitted Gaussian beam diffracts into *q*th orders (see labels of “−1st”, “0th”, and “+1st”), obeying the grating equation [9].

(1) $$n_{w}\times sin\theta_{q}=n_{\mathrm{air}}\times sin\theta_{i}+q\times \lambda /\Lambda$$

where $\theta_{q}$ stands for the angle of *q*th order relative to the y-axis (i.e., $\theta_{0}$ for 0th order), and $n_{w}$ and **$n_{air}$** are the RI of the inner core and ambient air, respectively. The produced diffraction coefficient is designated as $a_{q}$ ($\theta_{i}$) for the *q*th order, i.e., $a_{-1}$ ($\theta_{i}$), $a_{0}\left(\theta_{i}\right)$, and $a_{+1}\left(\theta_{i}\right)$. Here, the working principle can be intuitively understood as follows. The maximum coupling occurs in cases of normal incidences. With the help of grating structures, the *q*th-diffracted light at $\theta_{q}$ could be additionally coupled to the waveguide. Especially when $\theta_{q}$ turns to 0, the deflected light with a proportional power of $a_{q}$ turns to normal incidences, thereby leading to a solid enhancement of light-coupling efficiencies. As shown in Figure 1b, the binary gratings are occupied to actively tune diffracted angles and efficiencies. Resembling the experimental circumstances, other associated optical constants and geometry sizes in Figure 1 were applied.

## 3. Theoretical Model

**Figure 2:**
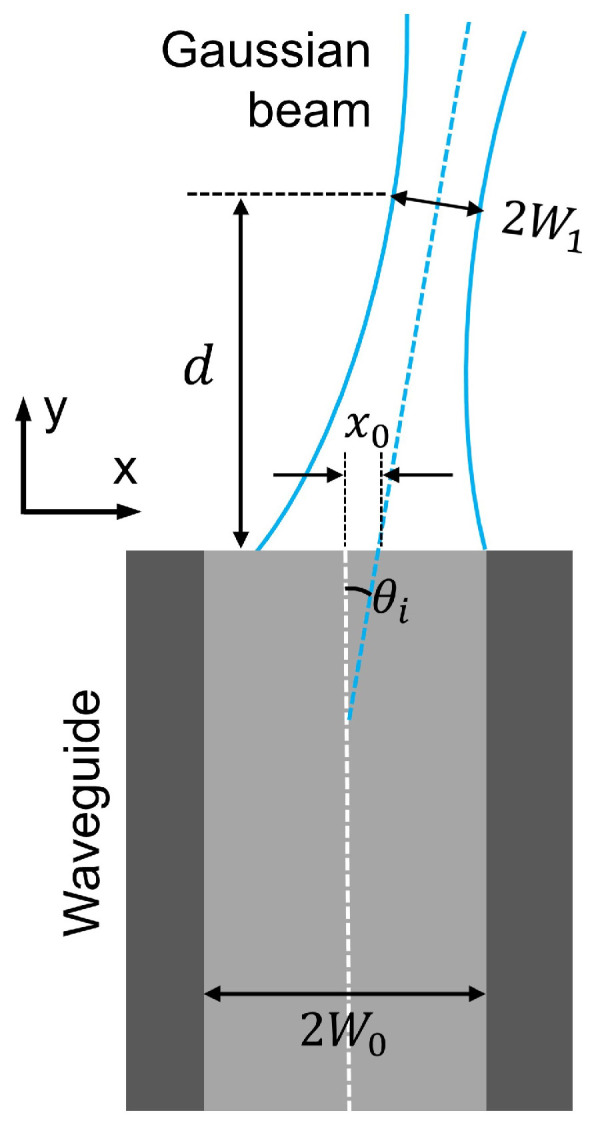
A sketch displaying a Gaussian beam coupled to a bare core-cladding waveguide.

The coupling efficiency between an input field and a waveguide has been explicitly examined in a multitude of literature [1,19,25,26]. In general, the efficiency η is related to the matching degree between the excitation and the waveguide fields. Here we focus on a typical condition of using an objective to collect light into waveguides. As explained in the work [25], the coupling efficiency $\eta_{b}$ of a 2D bare-facet model (see Figure 2, beam width: $2W_{1}$, core width: $2W_{0}$, incidence angle: $\theta_{i}$, working wavelength: λ) can be expressed by the following analytical equation:

(2) $$\eta_{b}\left(\theta_{i}\right)=\kappa \exp\left(-\kappa \left[\frac{x_{0}^{2}}{2}\left(\frac{1}{W_{1}^{2}}+\frac{1}{W_{0}^{2}}\right)+\frac{\pi^{2}\theta_{i}^{2}\left[W_{1}^{2}\left(d\right)+W_{0}^{2}\right]}{2\lambda^{2}}-\frac{x_{0}\theta_{i}d}{W_{1}^{2}}\right]\right)$$

where $\kappa =\left(4W_{1}^{2}W_{0}^{2}\right)⁄\left[{\left(W_{1}^{2}+W_{0}^{2}\right)}^{2}+\lambda^{2}d^{2}/\pi^{2})\right]$ and $W_{1}^{2}\left(d\right)=W_{1}^{2}\left[1+{\left(\lambda d/\pi W_{1}^{2}\right)}^{2}\right]$. $x_{0}$ and *d* indicate the beam-waveguide misalignment along x- and y-axes, respectively. All related parameters are plotted in Figure 2. Under the ideal arrangement (i.e., **d=0** and $x_{0}=0$), Equation (2) can be rewritten to

(3) $$\eta_{b}\left(\theta_{i}\right)=\kappa_{0}\exp\left(-\frac{2\pi^{2}{\theta_{i}}^{2}W_{1}^{2}W_{0}^{2}}{\lambda^{2}\left(W_{1}^{2}+W_{0}^{2}\right)}\right),\;\kappa_{0}=4/{\left(W_{0}/W_{1}+W_{1}/W_{0}\right)}^{2}$$

Taking grating effects into account, the corresponding coefficient $\eta_{g}$ becomes as in Equation (4).

(4) $$\eta_{g}\left(\theta_{i}\right)=\kappa_{0}\sum_{q}a_{q}\left(\theta_{i}\right)\exp\left(-\frac{2\pi^{2}\theta_{i}^{2}W_{1}^{2}W_{0}^{2}}{\lambda^{2}\left(W_{1}^{2}+W_{0}^{2}\right)}\right)$$

Equation (4) denotes the key analytical model where the waveguide coupling efficiency of η can be altered by the grating reinforcement. Using this model, we present the coupling efficiency of a grating-modified waveguide ($W_{0}=W_{1}=5\;\mu$m, $n_{air}=1.0$, $n_{g}=1.94$, $n_{w}=1.47$, λ=1.55μm, $a_{0}=a_{1}=0.5\times cos\theta_{i}$) in Figure 1c. Overall, there exists a significant efficiency increment in contrast to the bare waveguide. Besides the high efficiencies at normal incidences, two additional amplitude ridges (see labels of “1st” and “2nd”) emerge for the angle $\theta >20^{\circ }$, as the pitch increases from 1.1 to 3.15 μm. In particular, the lineshape of η becomes much flat due to the presence of the secondary diffracted orders (e.g., at a pitch > 2.5 μm). Here, η approaches the order of 10${}^{-1}$, and further improving η to a higher value (e.g., above 90%) is of great use for carefully arranging the diffraction efficiency $a_{q}\left(\theta_{i}\right)$.

## 4. Binary Coupling Grating

To devise proper geometries for wide-angle couplings, the binary gratings are explored to identify diffraction values. The core idea behind the later sections is to seek appropriate $a_{q}$ distributions as a function of $\theta_{q}$. Ideally, the gratings targeted for light couplings should possess the property where the maximum $a_{q}$ occurs in case of $\theta_{q}=0$. Honestly, to find the desirable $a_{q}\left(\theta_{q}\right)$ is rather challenging, since multiple factors (e.g., grating RI and geometries) could influence the diffraction coefficients [22,23,24]. Hence, this current report aims to demonstrate how grating could elevate coupling efficiency, and the comprehensive investigation for optimum grating-like structures would be a future issue.

**Figure 3:**
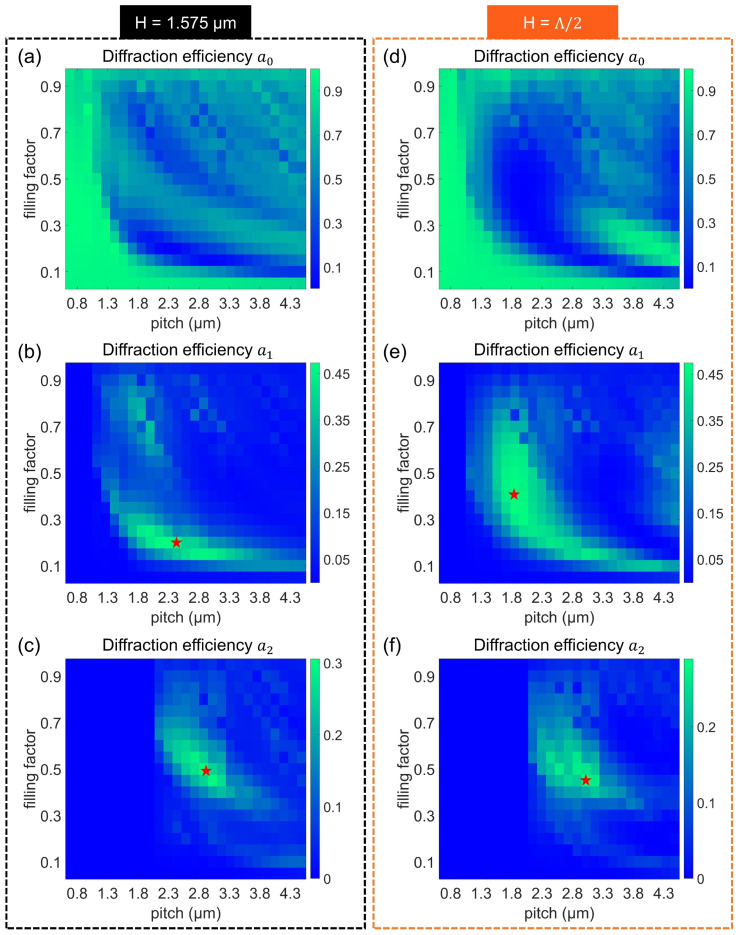
Diffraction efficiencies of binary gratings under normal incidences. Two vertical groups refer to the H at 1.575 μm and a half pitch, respectively. Horizontal pairs (**a**,**d**), (**b**,**e**), and (**c**,**f**) correspond to grating efficiencies of $a_{0}$, $a_{1}$, and $a_{2}$, separately. The red star-shaped markers in (**b**,**c**,**e**,**f**) are four gratings selected for the next studies.

We first swept grating parameters using normal incident light and then selected several shapes to inspect their diffraction efficiencies under varied-angle inputs. For speedy parameter-sweepings, a single grating structure packed with periodical boundaries was set up under plane wave excitations. The last waveguide-based full-sized simulation took the Gaussian-beam input in order to resemble actual experimental situations. Figure 3 provides color-coded graphs containing FEM-computed diffraction levels $a_{0}$, $a_{1}$, and $a_{2}$ (see detailed simulation models in Supplementary Materials). Each chart has two axes, with the x-axis being a pitch Λ (from 675 to 4500 nm by an interval of 150 nm) and the y-axis being a filling factor (defined as FF = w/Λ, with w being grating width in a single unit cell, ranging from 0.05 to 0.95 with a step of 0.05). The two vertical panels in black-dashed and orange-dashed frames represent the H values at a constant (H = 1575 nm) and a variable (H = Λ/2), respectively. Note that only the positive orders are displayed here, since the power ratios of binary gratings are symmetrically distributed.

In general, the two sets of graphs share high similarity. For instance, the enhancements of $a_{1}$ and $a_{2}$ could be captured in the green areas of Figure 3b,c,e,f. The increased $a_{1}$ and $a_{2}$ would reduce the value of $a_{0}$. This is because the sum efficiency of all associated orders should approach unity due to the lossless material used here. The maximum values of $a_{1}$ (between Figure 3b,d) and $a_{2}$ (between Figure 3c,e) are slightly diverse based on their configurations. Note that four geometrical arrangements with the highest values of either $a_{1}$ or $a_{2}$ (indicated by red stars) were intentionally picked for subsequent incident-angle sweepings.

## 5. Gratings under Large Inputs

**Figure 4:**
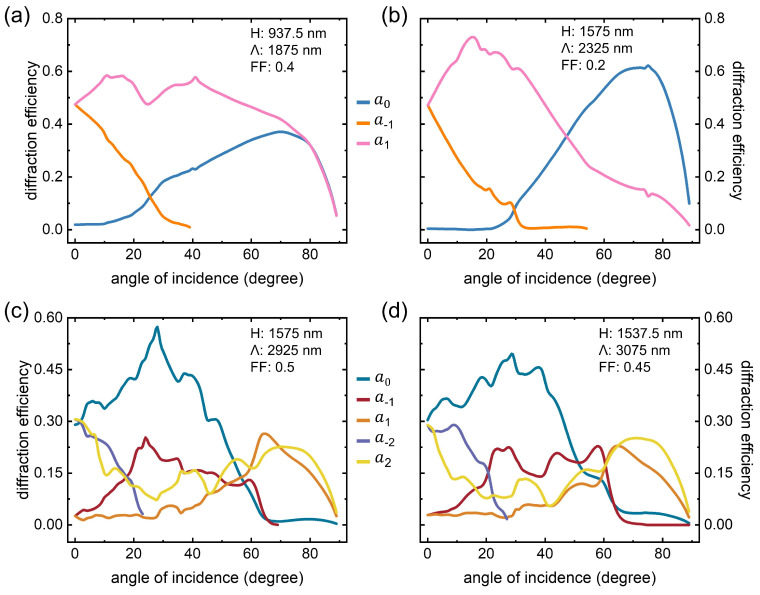
Binary grating diffraction efficiencies under varied incident angles. (**a**–**d**) Four parameter combinations, as values of H, Λ, and FF are suggested in the corner.

This section concentrates on the angle-changed diffraction efficiencies by employing four previously-chosen examples. The complete FEM-simulated results are plotted in Figure 4a–d, where their geometrical parameters (i.e., Λ, H, and FF) are listed in the corners. In terms of the smaller pitch (see the first row of Λ = 1875 and 2325 nm), merely $a_{0}$, $a_{1}$, and $a_{-1}$ orders are displayed here. In Figure 4a,b, both $a_{0}$ gradually increase to peaks and then drop to tiny numbers once the incoming angle is beyond $70^{\circ }$. In contrast, $a_{1}$ maintains a value plateau within certain scopes (i.e., (a): 0° to 60°, (b): $0^{\circ }$ to $25^{\circ }$), and it subsequently declines to be undetected. $a_{-1}$ quickly becomes zero, since it is physically absent for wide angles.

Regarding Figure 4c,d, the efficiency behaviors are rather complex. The larger pitch allows higher orders of $a_{\pm 2}$. Two $a_{0}$ arrive at their peaks (i.e., (c): 0.57 at $28^{\circ }$, (d): 0.49 at $29^{\circ }$), whereas all other orders of amplitude are below 0.35. Notably, there is a narrower adjustment space of $a_{1}$ compared to the mentioned cases in Figure 4a,b. The targeted $a_{2}$ fluctuates at around 0.15 across all angles, which may help to flatten the waveguides’ efficiency lineshapes.

## 6. Coupling Efficiency of Grating-Based Waveguides

**Figure 5:**
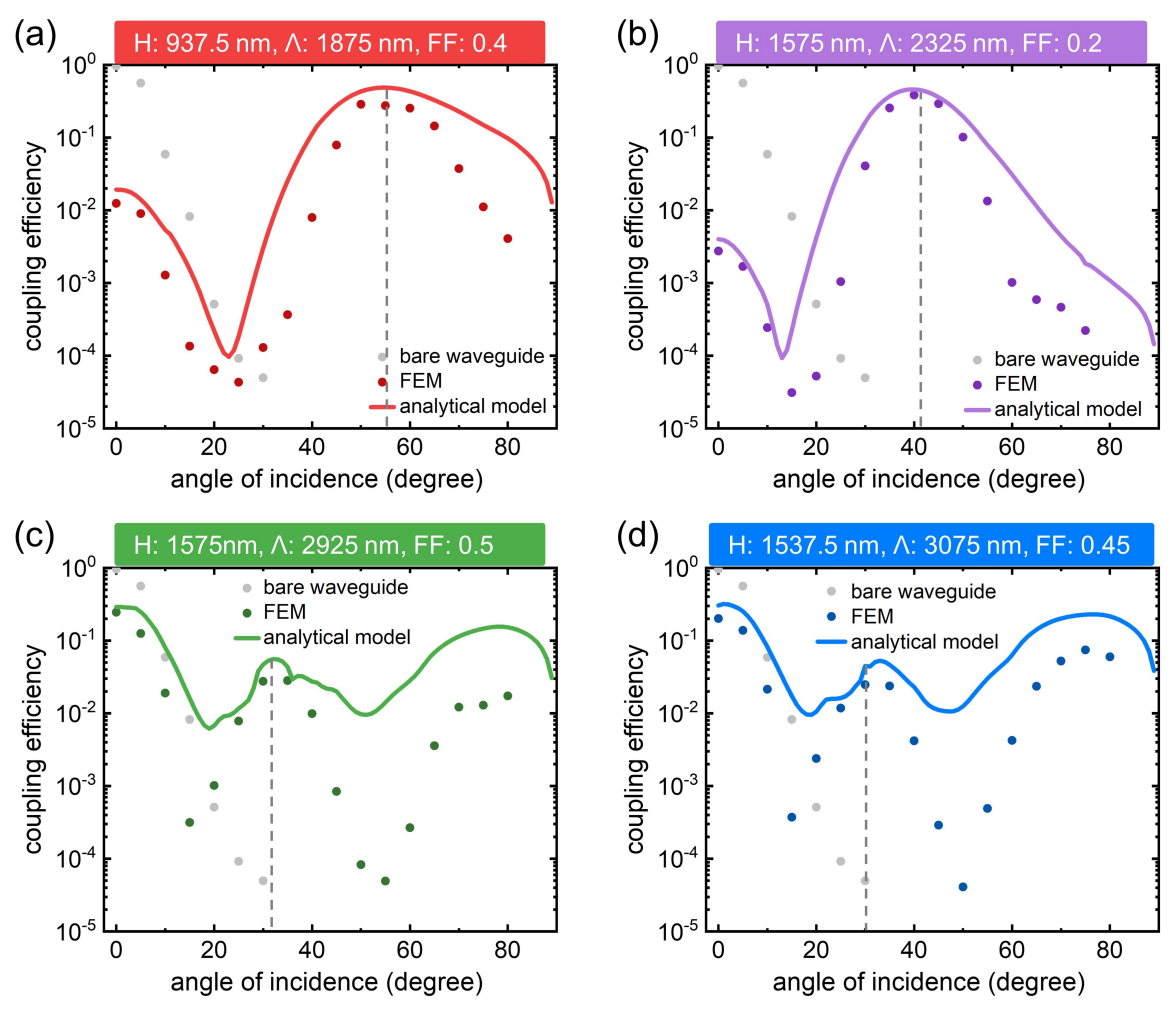
Coupling efficiencies of the grating-enabled waveguides computed by FEM (separated points) and the analytical model (solid lines). From (**a**–**d**), each graph relates to the grating configuration in Figure 4. The light gray dots and vertical dashed lines indicate the bare waveguide-coupled values and diffraction order angles, respectively.

Lastly, Figure 5 compares the coupling efficiencies of a grating-waveguide layout through the analytical model (see solid lines) and the FEM (see color dots, more information on waveguide simulation is provided in Supplementary Materials). Generally, the two approaches signify that the gratings would boost the waveguide light-coupling performances in contrast to the bare-endface case (indicated by the light-gray dots). Especially at the highlighted angles (marked by the vertical dashed lines), the two methods yield proximity amplitudes, both being lifted near the order of $10^{-1}$. The value differences are noted between the proposed analytical model and the FEM. Part of the reason can be attributed to the excitation discrepancy: the former values originate from periodical ports with plane waves, and the latter occupy Gaussian-beam inputs. In detail, Figure 5c,d displays two amplitude peaks, and there is only one value tip in Figure 5a,b. This is due to the grating pitch differences, where the larger periodical constant could impose higher-order transmissions.

For performance benchmarking, the coupling amplitudes of four cases together with sever-ring structure [19] are supplied in Table 1. Note that two major differences exist between our values and the original ones. On the one hand, we adopted the Gaussian beam excitation from [19], which considers the plane waves. On the other hand, we used the raw couplings data (i.e., $\eta \left(\theta_{i}\right)=P_{out}/P_{in}$), whereas the literature [19] has assessed the efficiencies normalized to the bare fiber (i.e., $\eta_{norm}\left(\theta_{i}\right)=\eta \left(\theta_{i}\right)/\eta \left(0\right)$). Hence, we conducted the numerical analysis on the seven-ring structure using the same FEM model (e.g., the ring patterns illuminated by focused Gaussian beams; see data in the table column “Seven-ring” and Supplementary Materials). It is noted that the 3D ring array is transferred as a 2D grating pattern, where the critical profile characteristics remain (i.e., H: 787.5 nm, Λ: 1.575 μm, FF: 0.5). Moreover, in terms of the large pitch Λ of 2928 and 3075 nm, two additional peaks are presented in Table 1. One is located at a smaller angle and another at a bigger angle.

As can be seen from the above table, the maximum η based on FEM was further lifted to 0.384 using a Λ = 1875 nm grating, almost 2.5 bigger than the counterparts of the seven-ring structure (i.e., 0.16). Although the current four designs may not be the best candidates for ideal couplings, they solidly validate that the amplitude could be further improved to, e.g., 50%, by incorporating well-designed patterns [27,28,29].

## 7. Conclusions

We reported a practical solution to improving waveguide broad-angle light-coupling abilities by tailoring the diffractive efficiency of the transmitted gratings. Firstly, the 2D analytical model, with a background of Gaussian-beam incidence on a waveguide, was concisely introduced, demonstrating that the coupling efficiency curves can be well-refined utilizing dielectric gratings. The follow-up search for proper binary shapes was carried out by FEM modelings. Four types of gratings were adopted for full-scale waveguide-based calculations after parameter-sweeping of their heights, pitches, and filling factors. Last, the results obtained from analytical predictions and FEM simulations were compared in detail, confirming the dramatic enhancement of η (up to 0.384, 2.5 times bigger than seven-ring patterned waveguides). In conclusion, our study provides a way to simulate waveguide’s light-coupling lineshape modified by grating effects. Future structural designs exploiting advanced algorithms [30] would help us determine the best-fit microscale geometries to satisfy the actual application demands.

## Figures and Tables

**Table 1:** Performance comparisons of coupling efficiencies improved by micro-structures.

Structure (Gaussian Beam Excitation)	Max η(θ)	
	Analytical Model	FEM
Seven-ring (Λ = 1575 nm)	N.A.	0.16 (70${}^{\circ }$)
Grating (Λ = 1875 nm)	0.485 (55${}^{\circ }$)	0.286 (50${}^{\circ }$)
Grating (Λ = 2325 nm)	0.459 (40${}^{\circ }$)	0.384 (40${}^{\circ }$)
Grating (Λ = 2925 nm)	0.055 (32${}^{\circ }$), 0.15 (77${}^{\circ }$)	0.028(35${}^{\circ }$), 0.017 (80${}^{\circ }$)
Grating (Λ = 3075 nm)	0.053 (33${}^{\circ }$), 0.23 (77${}^{\circ }$)	0.025 (30${}^{\circ }$), 0.074 (75${}^{\circ }$)

## Data Availability

Data are contained within the article or Supplementary Materials.

## Supplementary Materials

The following supporting information can be downloaded at: https://www.mdpi.com/article/10.3390/nano12223991/s1, Figure S1: Finite-element-method simulation models; Figure S2: Bare waveguide-based coupling efficiencies; Figure S3: The coupling efficiencies of a grating-enhanced waveguide; Figure S4: Two full-wave simulations for a grating-patterned waveguide. Figure S5: The electromagnetic field distributions of grating-configured waveguides.
