# Supplementary material for: Investigations on Grating-Enhanced Waveguides for Wide-Angle Light Couplings
Source: Nanomaterials (Basel). 2022 Nov 12;12(22):3991. doi: 10.3390/nano12223991 (PMC9698350; doi:10.3390/nano12223991)
Supplement: Supplementary file 1 [file nanomaterials-12-03991-s001.zip › nanomaterials-1994992-supplementary.pdf]

Supplementary Materials

Investigations on Grating-Enhanced Waveguides for Wide-Angle Light Couplings

Yitong Gu [1,2,†], Ning Wang [1,3,4,*,†], Haorui Shang [1,2], Fei Yu [1,2] and Lili Hu

1. Hangzhou Institute for Advanced Study, University of Chinese Academy of Sciences, No.1, Sub-Lane Xiangshan, Xihu District, Hangzhou 310024, China
2. Shanghai Institute of Optics and Fine Mechanics, Chinese Academy of Sciences, Shanghai 201800, China
3. Laboratory of Gravitational Wave Precision Measurement of Zhejiang Province, No.1, Sub-Lane Xiangshan, Xihu District, Hangzhou 310024, China
4. Taiji Laboratory for Gravitational Wave Universe, No.1, Sub-Lane Xiangshan, Xihu District, Hangzhou 310024, China

* Correspondence: wn@ucas.ac.cn
† These authors contributed equally to this work.

Section I: Finite-element-method (FEM) simulation models.

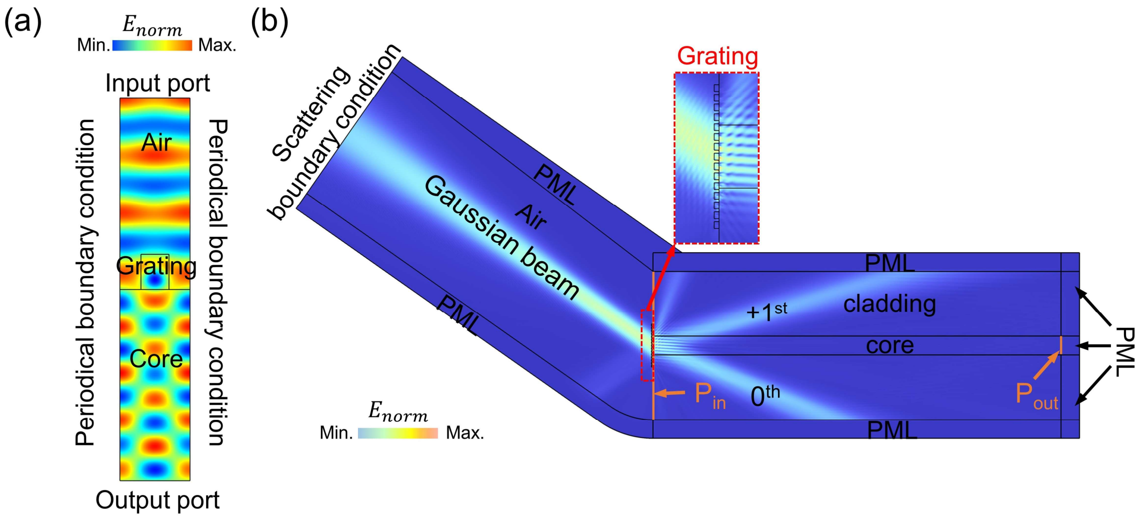

Figure. S1. The FEM models to analyze grating diffraction efficiency (a) and waveguide coupling efficiency (b).

Here two specific FEM models corresponding to grating diffraction efficiency and waveguide coupling efficiency are presented in Figure S1 (a) and (b), respectively. On the left, Figure S1 (a) depicts the grating model to compute its diffraction efficiency (see data in Figures 3&4). Overall a sandwich layout is applied with the top of the air layer (RI:1.0), middle of the grating (RI:1.94), and bottom of the waveguide core layer (RI:1.47). The periodical boundary conditions are configured to the adjacent cells. And two periodic ports, composed of upper excitation and lower output, are employed to calculate the diffraction efficiency at corresponding orders.

On the right, the 2D model consists of a focused Gaussian beam (tilted left section), dielectric gratings (highlighted in a dashed red frame), and the core-cladding waveguide (right section, R_{core} = 5.055 μm, $R_{cladding}$ = 50 μm.). The scattering boundary condition (left side) tunes beam parameters (e.g., width and focus length), and the incident angle is adjusted by rotating the beam direction. Except for the mentioned scattering boundary condition, the model is surrounded by perfectly matched layers (PMLs). The coupling efficiency η is defined as P_{out}/ P_{in}, where P_{out} and P_{in} are obtained by intensity integrations along corresponding ports (see orange lines). The RI of air, grating, core, and cladding is fixed at 1.0, 1.94, 1.47, and 1.446, respectively. Here, the incident light (working wavelength: 1550 nm) split into several orders (see labels of '0th' and '$+1^{st}$') due to the grating presence. Benefitting from the structure modification, the coupling efficiency η at large angles can be improved by collecting the qth-diffracted light.

Section II: Data comparison between the analytical model, FEM, and experiment.

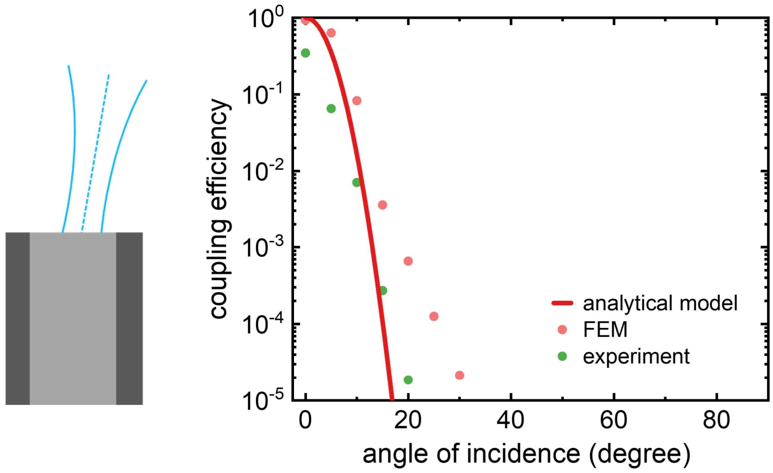

Figure. S2. Bare waveguide-based coupling efficiencies.

Figure S2 presents the bare fiber coupling efficiency based on the analytical model (solid line), numerical FEM simulations (light red dots), and actual measurement (green dots), respectively. The experiment is based on an SMF-28 fiber together with an aspherical lens (Thorlabs C560TME-C) and a laser (NKT SuperK compact, working wavelength: 1560 nm). In general, all three sets of values exhibit highly similar line shapes, where the coupling efficiencies drop to be undetected once the incidence angle exceeds around 20°.

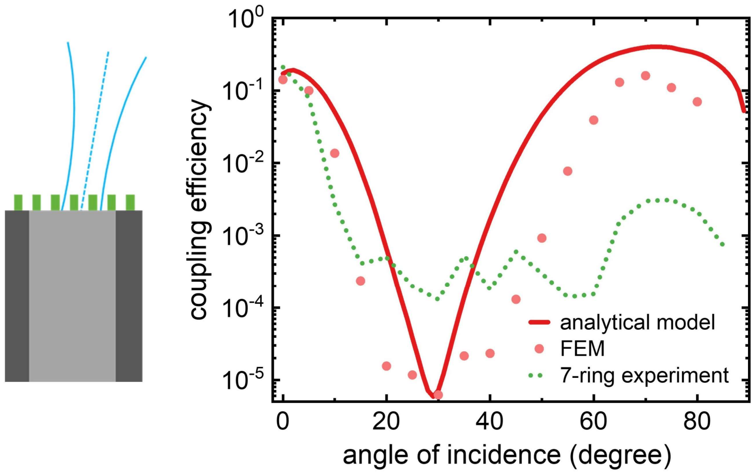

Figure. S3. The coupling efficiencies of a grating-enhanced waveguide.

Along with FEM and analytical data, we plot the grating-modified waveguide using information extracted from Ref.[ACS Photonics 2020, 7, 10, 2834–2841]. Note that there exist several differences between the simulations and experiments, as shown in Figure S3. The literature employs normalized efficiencies under plane wave excitations, while we simulate raw coupling data by Gaussian beam incidences. These factors influence the final line shape and lead to the value discrepancy. However, our value fits the general trend of measurements, i.e., improving η near the order of 10^{-1} at an angle of around 70°.

Section III: FEM models with varied incidence angles.

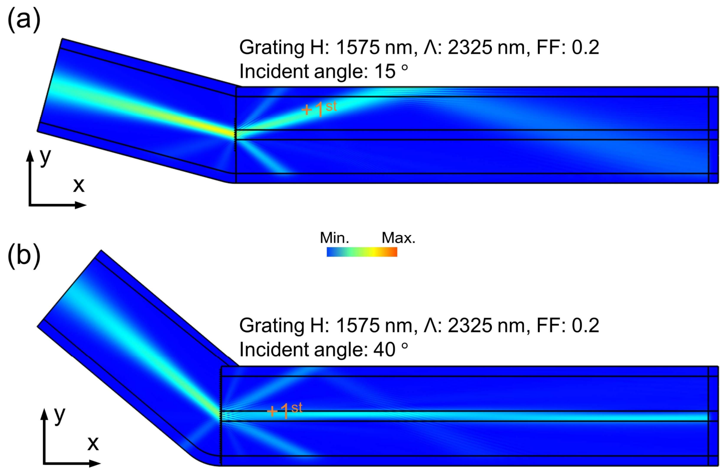

Figure S4. Two full-wave simulations for a grating-patterned waveguide under incidence angles of 15° (a) and 40° (b), respectively.

Figure S4 presents conditions where the incident light split into several orders due to the grating regulations. As can be seen from (a) and (b), the diffracted +1st light rotates as the incidence angle increases from 15 to 40 degrees. In (a), the +1st light is off the central waveguide x-axis, leading to the decoupled case. However, as highlighted in (b), the +1st beam is directly coupled into the core. More importantly, Figure S4 (b) reveals that +1st order light can be guided in the waveguide where the primary intensities are confined within the core area. This exactly illustrates the concept of using a grating structure to enhance light-gathering abilities.

Section IV: Electromagnetic field distributions of grating-waveguides.

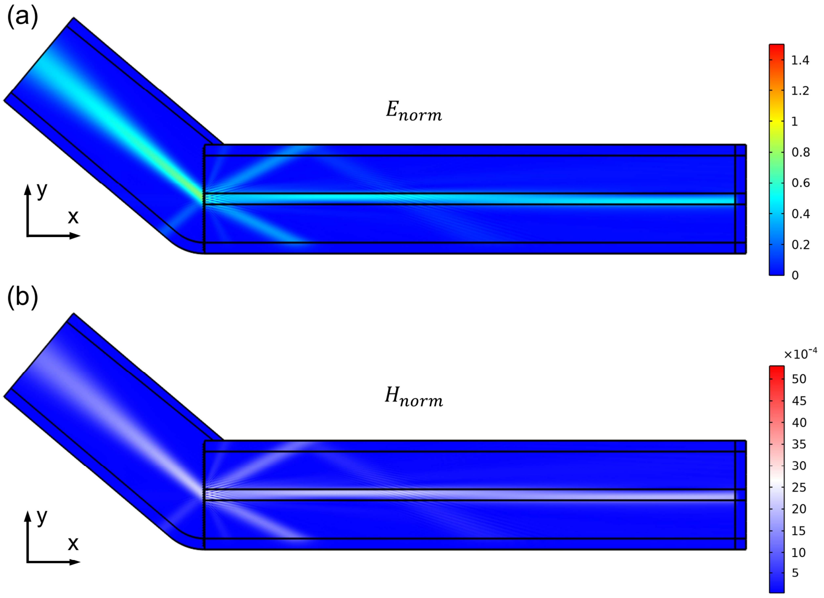

Figure S5. The electromagnetic field distributions of grating-configured waveguides.

Figure S5 (a) and (b) correspond to the E_{norm} and H_{norm} with the software-associated terms of $E_{norm} = \sqrt{E_x^2 + E_y^2 + E_z^2}$ and $H_{norm} = \sqrt{H_x^2 + H_y^2 + H_z^2})$, respectively. According to both images, the diffracted +1st beam couples into the waveguide and propagates along its core. The E_{norm} and H_{norm} are both well-confined within the core section. Two graphs further confirm that the diffracted light can be collected and transported by our models.
